# *Blastocystis sp.* in splenic cysts: causative agent or accidental association? A unique case report

**DOI:** 10.1186/1756-3305-7-207

**Published:** 2014-04-29

**Authors:** Helena Lúcia Carneiro Santos, Fernando Campos Sodré, Heloisa Werneck de Macedo

**Affiliations:** 1 Laboratório de Avaliação e Promoção da Saúde Ambiental, Instituto Oswaldo Cruz/FIOCRUZ, Av. Brasil, 4365–Pavilhão Lauro Travassos, Manguinhos, Rio de Janeiro, RJ 21.045-900, Brazil; 2 Departamento de Patologia, Universidade Federal Fluminense, Niterói, RJ, Brazil

**Keywords:** *Blastocystis* spp, Immunocompetent individual, Pathogenicity, Splenic cyst

## Abstract

**Background:**

*Blastocystis* sp. is one of the most prevalent parasites found in human stool and has been recently considered an opportunistic emerging pathogen in immunocompromised individuals. However, cases of invasive intestinal infections and skin rashes have been attributed to infection by *Blastocystis* sp in immunocompetent individuals, suggesting that it is an emerging parasite with pathogenic potential.

**Findings:**

We present a case of a 22 year old female patient who complained of pain in the left hypochondrium. Ultrasonography and abdominal computed tomography scans showed two splenic cysts. The c*yst fluid* analysis demonstrated numerous *Blastocystis* sp.; PCR and DNA sequencing analyses confirmed the presence of *Blastocystis* subtype 3.

**Conclusions:**

This is, to our knowledge, the first case report of the presence of *Blastocystis* subtype 3 in extra-intestinal organs and is strong evidence that *Blastocystis* sp. is potentially pathogenic and invasive. However, further studies are required to determine the pathogenicity of the parasite.

## Findings

*Blastocystis* spp. are parasites of the intestinal tract found in many hosts including humans [1]. This pathogen is commonly found in apparently healthy and asymptomatic individuals and in patients with gastrointestinal disease. Its pathogenicity has been reported in the literature in immunocompromised pediatric, cancer, and HIV-infected patients [2-5], however, the clinical relevance of *Blastocystis* sp. in immunocompetent individuals remains unclear. The association between *Blastocystis* sp. and arthritis, dermatological disorders, and irritable bowel syndrome [6-8] have also been reported. In addition, its invasive potential has been suggested in animal models [9,10] and in humans [11-13]. Recently, cases of enteroinvasion by *Blastocystis* sp were shown *in vivo* through endoscopy and biopsy analyses [13]. In this case report, *Blastocystis* sp. was detected in ulcers in the cecum, transverse colon, and rectum of an immunocompetent patient.

*Blastocystis* has been traditionally named *Blastocystis hominis* when isolated from human fecal material*s*. However, recent phylogenetic analyses suggest limiting its name to “*Blastocystis sp*ecies” because of their genetic diversity. This parasite has been considered as a species complex comprising 13 subtypes, of which at least nine have been found in humans [14]. Furthermore, they exhibit wide genetic diversity that is sufficient to assign them to different species [15]. The confirmation of their species status and determination of virulence and pathogenic profiles might explain why some patients are asymptomatic while others present clinical symptoms [16].

*Blastocystis* spp. was discovered over a century ago, however, many issues regarding its infection still remain unanswered. Accumulating evidence reinforces the pathogenic potential of *Blastocystis sp*. in immunocompetent individuals [6-8]; however, systematic studies characterizing different clinical isolates of *Blastocystis* subtypes and new diagnostic approaches are needed to improve our understanding about these cases. This is the first case report describing the presence of *Blastocystis* sp. in the fluid of splenic cysts. According to our observation, the following question is raised: ‘Can *Blastocystis* be the culprit for the formation of splenic cysts or is this association based on other reasons?’

### Case presentation

**Figure 1:**
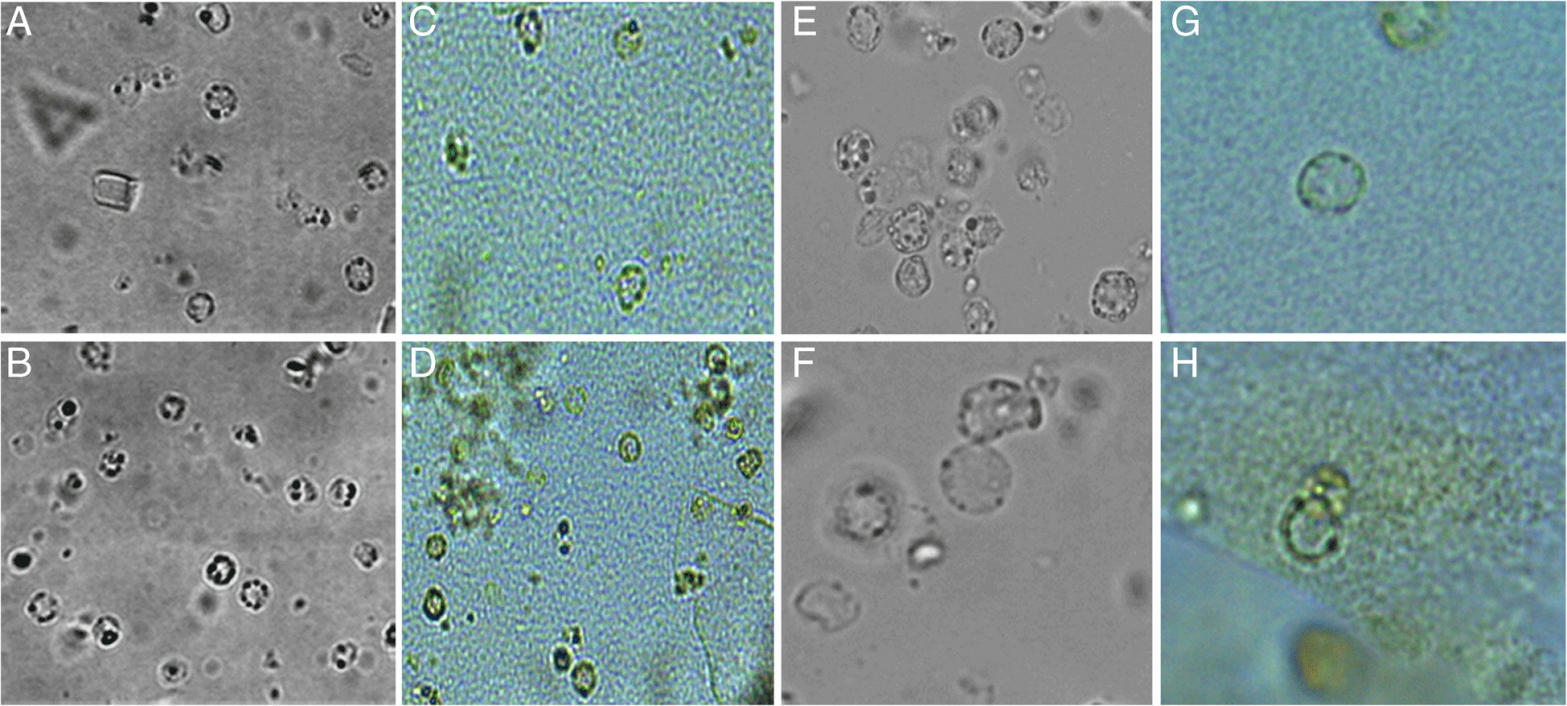
**Light microscope images of *Blastocystis *sp. in splenic fluid specimens. A** and **B** (unstained wet slides), **C** and **D** (iodine stained wet slides), 40× magnification. **E** and **F** (unstained wet slides), **G** and **H** (iodine stained wet slides), 100× magnification.

**Figure 2:**
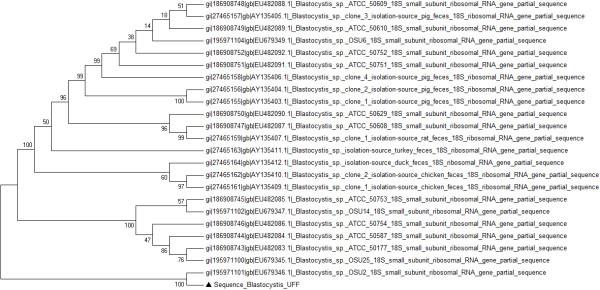
**Phylogenetic analysis of partial SSU rRNA sequences of *Blastocystis *isolates.** Neighbor-Joining tree displaying the relationships of *Blastocystis* isolates, inferred by analysis of partial SSU rDNA sequence data using Kimura’s 2 parameter distance estimates. The number at each branch point represents the percentage of bootstrap support calculated from 1,000 trees. The sequences used for comparison were from Genbank. The triangle indicates isolates from this study.

A 22 year-old white female, residing in Niteroi, Rio de Janeiro State, Brazil, sought treatment in 2009 reporting localized pain in the left hypochondrium. The ultrasonography showed a 15.1 × 11.1 cm cyst in the spleen. In 2012, this patient returned for medical attention due to an intensification of the pain that affected her walking. An abdominal computed tomography (CT) scan revealed the presence of two spleen cysts (11 × 11 cm) with peripheral calcifications (one intra- and one extra-splenic). The hematological and biochemical tests showed results within normal limits and the chest *x*-ray was normal, however, the urine culture was positive for *Streptococcus agalactiae*. Surgical treatment was recommended and, one month prior to operation, the patient received the pneumococcal *Haemophilus influenzae* and meningococcal vaccines. A laparoscopic excision of the splenic cyst was performed and the postoperative recovery was uneventful; the patient was discharged on the second post-operative day. The cytological examination of cyst fluid showed the presence of histiocytes, lymphocytes, polymorphonuclear leukocytes, and lysed erythrocytes. No evidence of cancerous cells was observed and the biochemistry results were as follows: 3.6 g/dl albumin, 3 mg/dl glucose, 137 mEq/L sodium chloride, 3.9 Eq/L potassium chloride, 1132 U/L lactate dehydrogenase (LDH), 76 U/L amylase, and 65 U/L lypase. Furthermore, the presence of crystals and *Blastocystis* sp. were observed in the microscopic examinations of the fluid contained in the cyst (Figure 1). The presence of *Blastocystis sp*. was further confirmed by PCR using primers and conditions previously described [17] and subsequent sequencing. The obtained small subunit *ribosomal DNA* nucleotide partial sequence was compared to other sequences available in this database using the BLASTN program from the National Center for Biotechnology Information (NCBI) server (http://www.ncbi.nlm.nih.gov/BLAST). This analysis showed 99% similarity between our sequence and the *Blastocystis* sp. OSU2 sequence (GenBank: EU679346) characterized as *Blastocystis* subtype 3. The phylogenetic analysis showed that sequence of this study clustered with *Blastocystis* sp OSU2 sequence (Figure 2).

### Discussion

Splenic cysts constitute very rare clinical entities. They may occur secondary to trauma or parasitic infestations, particularly by *Echinococcus granulosus*[18,19]. Cases of isolated splenic involvement in hydatid disease are not very frequent even in endemic regions [18]. Interestingly, this report describes the presence of *Blastocystis* subtype 3 in splenic cysts, a parasite mostly found in human stool. A previous case study described *Blastocystis* present in the liver abscess aspirate of a patient with history of fever, watery diarrhea and high Anti-*Entamoeba histolytica* antibody titer [11]. Although the pathogenic mechanisms are unclear; we speculate that two hypotheses could be considered to explain how the *Blastocystis* sp got into the spleen. First, *Blastocystis* sp might penetrate *and* invade the intestinal mucosa and submucosa causing ulcers, and progress through the blood and/or lymphatic system and migrate to the spleen. Second, the parasite might gain access to extra-intestinal sites with the help of coinfections or other pathological circumstances. To complement these hypotheses, *Blastocystis* sp have to survive during infection, essentially by responding rapidly to changes in the microenvironment in intestine, blood and escaping host defense mechanisms. There are few studies addressing the mechanism of pathogenesis, regarding cellular microbiology, immune evasion, and life cycle of *Blastocystis* sp. This parasite one of the most difficult organisms to identify in stool samples because of their morphological biodiversity; some of the commonly reported forms in culture or in fecal specimens are vacuolar, granular or amoeboid. However, other forms that might occur with relative frequency might be missed by untrained examiners [1]. Conversely, the lack of standardized diagnosis may also lead to misinterpretation of results [1]. Recently, a study revealed poor agreement in reporting *Blastocystis* sp. positive specimens when comparing the diagnostic performance of various European reference laboratories [20]. Several molecular epidemiological studies suggest a possible correlation between subtypes and clinical presentations of *Blastocystis* infections. Other studies observed no association between presence of this organism and disease [21,22]. These discrepant results might be explained by subtypes with differences in virulence, or by low sensitivity in the diagnosis techniques used. This scenario is strikingly similar to that of an erroneous diagnosis of *Entamoeba histolytica*. For many years, the virulence and pathogenicity of *E. histolytica* was questioned until molecular techniques irrefutably showed that there are two genetically distinct, but morphologically identical, species in what was formerly known as *E. histolytica*. Differences in the pathogenesis of *E. histolytica* and *E. dispar* also helped explain the epidemiology, presentation of symptoms, and pathology of amoebiasis [23].

Currently, there is not enough evidence showing that *Blastocystis* sp. is a nonpathogenic organism and its association with gastrointestinal diseases raises questions about its pathogenicity [5-8]. Moreover, there are accumulating data suggesting its pathogenic potential in immunocompetent individuals [1,12-14].

The genome of *Blastocystis* subtype 7 encodes proteases, hexose digestion enzymes, lectins, protease inhibitors, and glycosyltransferases besides several proteins that are predicted to be secreted [24]. The roles of some of these proteins are known in other parasites [25] with direct connections to their pathogenicity in processes such as host cell invasion, excystation, metabolism, cytoadherence, and other virulence functions [24]. Thus, proteomics and transcriptomic analyses will be useful in order to show whether these predicted proteins have any role in the pathogenesis of *Blastocystis*.

Protease activity has been described in *Blastocystis* spp. isolated from symptomatic patients [26-28]. In addition, other studies have demonstrated that cysteine proteases from *Blastocystis* can increase epithelial permeability by modulating the tight junction complex [29], induce pro-inflammatory cytokine interleukin-8 (IL-8) [30], and degrade human immunoglobulin A (IgA) [31]. Cysteine proteases are important enzymes for host invasion and infection and are well recognized as virulence factors in pathogenic protozoa [25].

In this study, *Blastocystis* subtype 3 was detected in splenic cysts. The literature on the molecular analysis of human *Blastocystis* isolates suggests that they are mostly of genotype subtype 3. Genotype variability has been reported to play an influential role in the pathogenicity of *Blastocystis*[32,33]. However, previous studies have associated subtypes 1, 4, and 7 with human pathology, whereas subtypes 2 and 3 predominate among healthy carriers [12]. Infections with mixed subtypes, and the high degree of genetic diversity among subtypes, obscure possible correlations between pathogenicity and *Blastocystis* subtypes [5,34,35].

### Conclusions

To our knowledge, this is the first report describing *Blastocystis* subtype 3 in an extra-intestinal organ. We have no knowledge as to how the parasite gained access to the spleen. The answer to this question will deepen our understanding about the pathogenicity of *Blastocystis* sp. Its pathogenic potential is a relevant threat to immunocompetent individuals and this report emphasizes the importance of an increased awareness and recognition of this pathogen.

## Consent

Written informed consent was obtained from the patient for publication of this Case report. A copy of the written consent is available for review by the Editor of this journal.

## Competing interests

The authors declare that they have no competing interests.

## Authors’ contributions

HLCS, FCS and HWM conceived and designed the study. FCS carried out the microscopy examination. HLCS carried out the molecular approaches, data analysis and interpretation, and manuscript writing. HWM supervised the study, carried out the laboratory work, intellectual interpretation and critical. All authors read and approved the final version of the manuscript.
